# Biodiversity conservation values of fragmented communally reserved forests, managed by indigenous people, in a human-modified landscape in Borneo

**DOI:** 10.1371/journal.pone.0187273

**Published:** 2017-11-29

**Authors:** Yayoi Takeuchi, Ryoji Soda, Bibian Diway, Tinjan ak. Kuda, Michiko Nakagawa, Hidetoshi Nagamasu, Tohru Nakashizuka

**Affiliations:** 1 Center for Environmental Biology and Ecosystem Studies, National Institute for Environmental Studies, Onogawa, Tsukuba, Japan; 2 Faculty of Literature and Human Sciences, Osaka City University, Sugimoto, Sumiyoshi, Osaka, Japan; 3 Botanical Research Centre Semenggoh, Sarawak Forestry Corporation, Kuching, Sarawak, Malaysia; 4 Graduate School of Bioagricultural Sciences, Nagoya University, Nagoya, Japan; 5 The Kyoto University Museum, Kyoto University, Sakyo-ku, Kyoto, Japan; 6 Graduate School of Life Sciences, Tohoku University, Sendai, Japan; Chinese Academy of Forestry, CHINA

## Abstract

This study explored the conservation values of communally reserved forests (CRFs), which local indigenous communities deliberately preserve within their area of shifting cultivation. In the current landscape of rural Borneo, CRFs are the only option for conservation because other forested areas have already been logged or transformed into plantations. By analyzing their alpha and beta diversity, we investigated how these forests can contribute to restore regional biodiversity. Although CRFs were fragmented and some had been disturbed in the past, their tree species diversity was high and equivalent to that of primary forests. The species composition of intact forests and forests disturbed in the past did not differ clearly, which indicates that past logging was not intensive. All CRFs contained unique and endangered species, which are on the IUCN Red List, Sarawak protected plants, or both. On the other hand, the forest size structure differed between disturbed and intact CRFs, with the disturbed CRFs consisting of relatively smaller trees. Although the beta diversity among CRFs was also high, we found a high contribution of species replacement (turnover), but not of richness difference, in the total beta diversity. This suggests that all CRFs have a conservation value for restoring the overall regional biodiversity. Therefore, for maintaining the regional species diversity and endangered species, it would be suitable to design a conservation target into all CRFs.

## Introduction

Biodiversity has been declining because of forest loss and degradation by human developments and activities [1], particularly in the tropical regions [2–5]. Globally, much attention is paid to conservation strategies that integrate traditional indigenous practice, as observed in Article 8(j) of the Convention of Biological Diversity [6]. It has been acknowledged that traditional and local knowledge and practices contribute to biodiversity conservation [7–10]. In Asia, traditional forest management systems can be commonly found, such as “culturally protected forests” in China [8], “community forests” in Nepal [11], and “sacred forest grooves” in India [12]. These forests provide essential ecosystem services, such as drinking water, area for graves, recreation, hunting, plant gathering, and ritual/spiritual use, to local people. However, these traditional uses and customs are threatened because of urbanization and development [13]. Thus, conservation strategies that incorporate traditional practice can function as a *safety net* to minimize the loss of traditional knowledge and practice in the region as well as biodiversity.

In rural areas in Borneo, extensive logging started since the 1970s, and most forests have been logged at least once. In the current landscape, forested areas have transformed into plantations for oil palm and acacia. Only small patches of forest remain that the local indigenous Iban communities deliberately preserve within their area of shifting cultivation [14]. We define this traditionally managed forest as communally reserved forests (CRFs), which are customarily reserved forests at a community level for cultural and practical use (i.e., provisioning and cultural service). A CRF is called a “*pulau*” in Iban language, which literally means “island,” because CRFs are unevenly distributed and surrounded by fallow or secondary forests. It was from these reserves that villagers obtained various kinds of natural resources, such as wood for construction of houses and boats, and canes [15]. CRFs are generally supposed to be untouched timber sources, with few trees having been harvested from most CRFs [7, 16], except for an emergency such as fire. Therefore, they can have a primary forest-based biodiversity and function as reservoirs of biodiversity in the region.

However, in the current landscape, CRFs are fragmented and isolated from other primary forests. Forest fragmentation leads to a decline in local and regional diversity, particularly the loss of rare and endangered species, because of edge and isolation effects [17–19] and a subsequent homogenization of the species composition [20]. To demonstrate that CRFs are adequate targets for regional biodiversity conservation, both 1) alpha diversity of each CRF, including the existence of rare species, and 2) beta diversity, which is a key component of regional (gamma) diversity that accumulates inter-site differences between local species assemblages (alpha diversity) need to be assessed. In particular, beta diversity can directly assist conservation planning because it represents underlying ecological processes of diversity maintenance of the biological species assemblage [21]. For example, directional changes of species composition along a spatial or environmental gradient (i.e., the turnover or distance-decay of similarities) will indicate if multiple forest patches with such a variation would be necessary. In addition, the nestedness of the species composition or high richness differences among patches imply that conservation efforts should focus on sites with a high diversity, rather than species-poor sites [21]. The beta diversity index consist of two components, species replacement and richness difference [22]. This approach enables us to understand the relative importance of the mechanisms that influence the beta diversity and gives us suggestions for conservation strategies.

This study focused on CRFs and explored the conservation values of CRFs that have been managed by local communities in Sarawak, Malaysia, in terms of their alpha and beta diversity. First, we investigated the alpha diversity of each CRF in terms of their endangered species and species richness (*S*). We also investigated whether multiple CRFs contribute to the regional species diversity index. Furthermore, we investigated the relative contributions of replacement and richness, by dividing the beta diversity index into these components and characterizing their relative importance in conserving the species diversity in this region. Finally, we discuss if CRFs retain a conservation value in the region, including the social aspects of conservation.

## Materials and methods

### Study site

**Fig 1:**
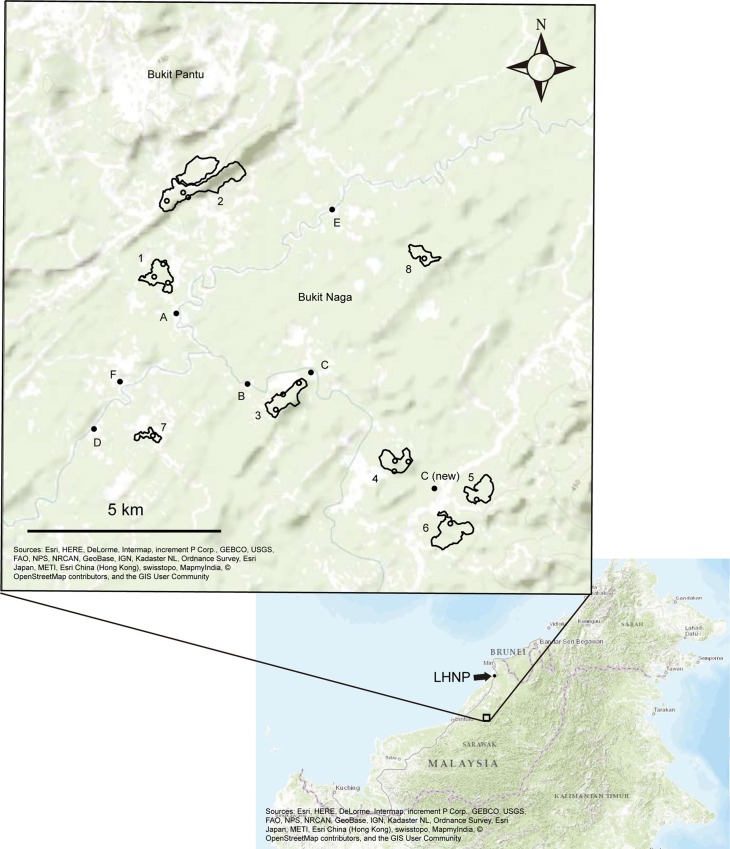
Map of the targeted study area in jelalong. The letters indicate the targeted villages except for F, where does not have a CRF. Village C was moving to their longhouse into the location C (new). The numbers indicate the communally reserved forests (CRFs), and the open circles in CRFs are the studied plots and the closed ones are villages. The location of the Lambir Hills National Park (LHNP) is shown in the lower map.

This study was conducted in the central part of the Jelalong River basin, Sarawak, Malaysia (Fig 1). The area comprises various types of landscapes, such as primary mixed dipterocarp forest (MDF), *kerangas* forest with nutrient-poor soils (podzol), logged forest, fallow forest, paddy fields, and acacia or oil palm plantations. The forest (primary and logged forest) cover in the area (ca. 700km^2^) in 2015 has been estimated to be approximately 50% (Takeuchi et al., unpublished data). The main driving force of land use change here is oil palm plantations. This mixed landscape was considered appropriate for examining the conservation value of remnant CRFs in the developing area. The average annual rainfall during 2006–2012 in Tubau (3°9′N, 113°42′E), which is located approximately 5 km from the study area, was 4,556 mm. The mean air temperature during 2006–2012 at Bintulu airport (3°7′N, 113°1′E, approximately 85 km from the study area) was 26.8°C. The elevation in the study area ranges from 30 to 200 m above sea level. The research was conducted under the permit issued by Forest Department Sarawak. We also got permit to conduct the research from the headmen of local villages in the study area.

The study area comprises six villages with local Iban and Penan people. Traditionally, they have been engaged in slash-and-burn rice cultivation and rubber tapping since the 1960s. More recently, they have begun to plant oil palms on a small scale as new cash crops. Five out of these six villages have at least one CRF, and in five villages, there are ten CRFs in total. In this study, we investigated eight out of these ten CRFs because one was supposed to be transformed into an oil palm plantation, whereas another was hard to access. The most important factor for villages that possessed at least one CRF was a water catchment forest, from which water pipes were laid to the settlements (longhouse). CRFs used as a drinking water resource are called *pulau paip* (pipe) or *pulau ai* (water) in Iban language. Although a CRF is supposed to be an intact or untouched forest [14], recent CRFs in this area contain rather disturbed forest patches. Six out of the eight CRFs experienced selective logging at least once in the past although it was not clear cut (Table 1). All CRFs are used by villagers for hunting and for collecting non-timber forest products, such as fruits, vegetables, mushrooms, bamboo, rattan, resin, and wild rubber. Most of those products from CRFs are used for consumption. However, sometimes villagers gain cash income by selling these products, such as rattan baskets (village A).

**Table 1:** Diversity indices and size structure of CRFs. The species richness (*S*) and Shannon diversity index (*H*) were standardized for the plot area.

Village	A		B	C			D	E	Lambir Hills NP		
Ethnic group	Iban		Penan/Iban	Iban			Penan/Iban	Iban			
CRF no.	1	2	3	4	5	6	7	8	4ha		8ha
**Area (ha)**	36	125	38	32	48	32	10	20	~7000		
**No. of plots**	3	3	3	3	1	1	1	1			
**Water catchment**		✔	✔			✔^†^	✔	✔			
**Disturbance**	✔			✔	✔	✔	✔	✔			
**Last cutting**	1950s			1980s	2007	1980s	1980s	1980s			
**Diversity indices per 0.25 ha plot**											
***S* (± SD)**	43.0 ± 8.7	88.0 ±11.5	80.3 ± 6.8	84.0 ± 1.7	79.0	89.0	97.0	74.0	91.5 ± 8.7	84.4 ± 18.4	
***H* (± SD)**	3.1 ± 0.4	4.1 ± 0.3	4.2 ± 0.2	4.0 ± 0.1	3.9	4.2	4.1	4.0	4.3 ± 0.1	4.1 ± 0.3	
**Size structure**											
Proportion (%) of DBH >50cm*	6.9 ^ABC^	10.8 ^A^	8.1 ^AB^	3.6 ^CD^	2.3 ^CD^	3.9 ^BCD^	1.0 ^D^	6.0 ^ABC^	6.4 ^B^	6.3 ^BC^	
**Total basal area (m**^**2**^**/ha)**	37.1	43.7	36.8	34.3	32.1	34.6	23.3	29.4	36.4	34.6	
**Maximum size of DBH (cm)**	90.5	122.5	160.0	98.5	71	66.9	57.4	73.5	146.4	207.4	

†The CRF is established to be used as a water catchment forest for the new longhouse of the village.

*Proportion followed by the same letter is not significantly different at a level of significance of 0.05 by pairwise Fisher’s test adjusted using false discovery rate (Benjamini & Hochberg, 1995, J R Stat Soc Series B Stat Methodol, 57(1):289–300.).

The study area is approximately 100 km from the Lambir Hills National Park, Miri, Sarawak (LHNP, 4°08′–4°12′N, 114°00′–114°07′E, 20–220 m above sea level, Fig 1), which contains a typical primary lowland MDF. We used data obtained in 2000 from two permanent vegetation plots in LHNP (4 and 8 ha, respectively) as the control plots of primary forest.

### Biodiversity assessment of CRFs

#### Tree census

In October 2013 and February 2014, we established 16 plots of 50 m × 50 m each (0.25 ha) in the eight CRFs (Fig 1). Six CRFs had been logged approximately 30 years before (Table 1). In each plot, all tree stems with a diameter at breast height (DBH, at 1.3 m above ground) > 10 cm were tagged, identified to species level based on vegetative samples, and their DBH measured. Voucher specimens were stored in the Botanical Research Centre Sarawak, Malaysia.

#### Data analysis–alpha diversity and community structure

To evaluate the alpha diversity in each CRF, the number of species (*S*) and Shannon diversity index (*H*) were calculated. In addition, the rarefaction curves (i.e., the expected *S* in random subsamples of a given sample size from the focal community in a fixed area) were determined for the number of trees in each plot. This was performed to determine the sample size or area effect of each forest, using the function “rarefy”, which provides rarefaction of species richness in each sample size, from the package vegan in R [23]. To standardize the plot sizes, 1000-times re-sampled 0.25-ha areas within the plots in LHNP were averaged to describe the rarefaction curves. We also averaged the result for three plots in CRFs 1–4. We used LHNP data to compare CRF values with primary forest values. Because we used 4- and 8-ha plot data from LHNP, we standardized the plot area. To determine the rarefaction curve, we randomly re-sampled a 0.25-ha area for 1000 iterations and calculated the 95% confidence intervals. The similarity in species community composition between the forests was illustrated by a non-metric multidimensional scaling (NMDS, [24]). NMDS calculates an ordination based on a similarity matrix, using Bray–Curtis distances. We compared data from 16 plots of eight CRFs in the Jelalong area with two plots from LHNP. Here, we used all data of the LHNP plots as standardization would result in poor performance of the NMDS probably because of the smaller sample size. NMDS was calculated with the function metaMDS in the R package “vegan” using species level data.

#### Data analysis–beta diversity

First, the cumulative species–area curves were produced, to examine differences in the contribution to regional diversity between smaller and larger CRFs [25, 26]. We plotted the cumulative number of species against the number of plots added and the cumulative area of CRFs by sorting the patches from small to large and from large to small.

Second, two dissimilarity indices, the abundance-based (Bray-Curtis index, *D*_*B*_) and presence-absence index (Jaccard index, *D*_*J*_), were calculated for all paired comparisons of CRFs. Correlations between dissimilarity and geographic distances were examined with a Mantel tests (using the R package “ecodist”). Furthermore, to investigate whether variations in beta diversity were caused by *S* differences among CRFs or by species replacement (turnover), we employed the method of Legendre [22]. The total beta diversity (*BD*_*Total*_) is calculated as

$${BD}_{Total}=\sum_{h=1}^{n-1}\sum_{i=h+1}^{n}\frac{D_{hi}^{}}{n(n-1)}$$,

where *D*_*hi*_ represents the dissimilarity between sites *h* and *i*. As *D*_*hi*_ can be decomposed into two factors, replacement (*Repl*_*hi*_) and richness difference (*RichDiff*_*hi*_), the relationship can be written as follows:

$${BD}_{Total}={Repl}_{Total}+{RichDiff}_{Total}$$,

where ${Repl}_{Total}=\sum_{h=1}^{n-1}\sum_{i=h+1}^{n}\frac{{Repl}_{hi}^{}}{n(n-1)}$

and

$${RichDiff}_{Total}=\sum_{h=1}^{n-1}\sum_{i=h+1}^{n}\frac{{RichDiff}_{hi}}{n(n-1)}.$$

This relationship allows us to calculate the proportion of *BD*_*Total*_ accounted for by the replacement and richness difference fractions as follows:

$${Repl}_{Prop}=\frac{{Repl}_{Total}}{{BD}_{Total}}\;and$$

$${RichDiff}_{Prop}=\frac{{RichDiff}_{Total}}{{BD}_{Total}}.$$

We subsequently produced the triangular graphs to represent the pairwise indices 1 − *D*_*hi*_, *Repl*_*hi*_, and *RichDiff*_*hi*_ which also followed the method of Legendre [22].

## Results

### Tree species composition and diversity

**Fig 2:**
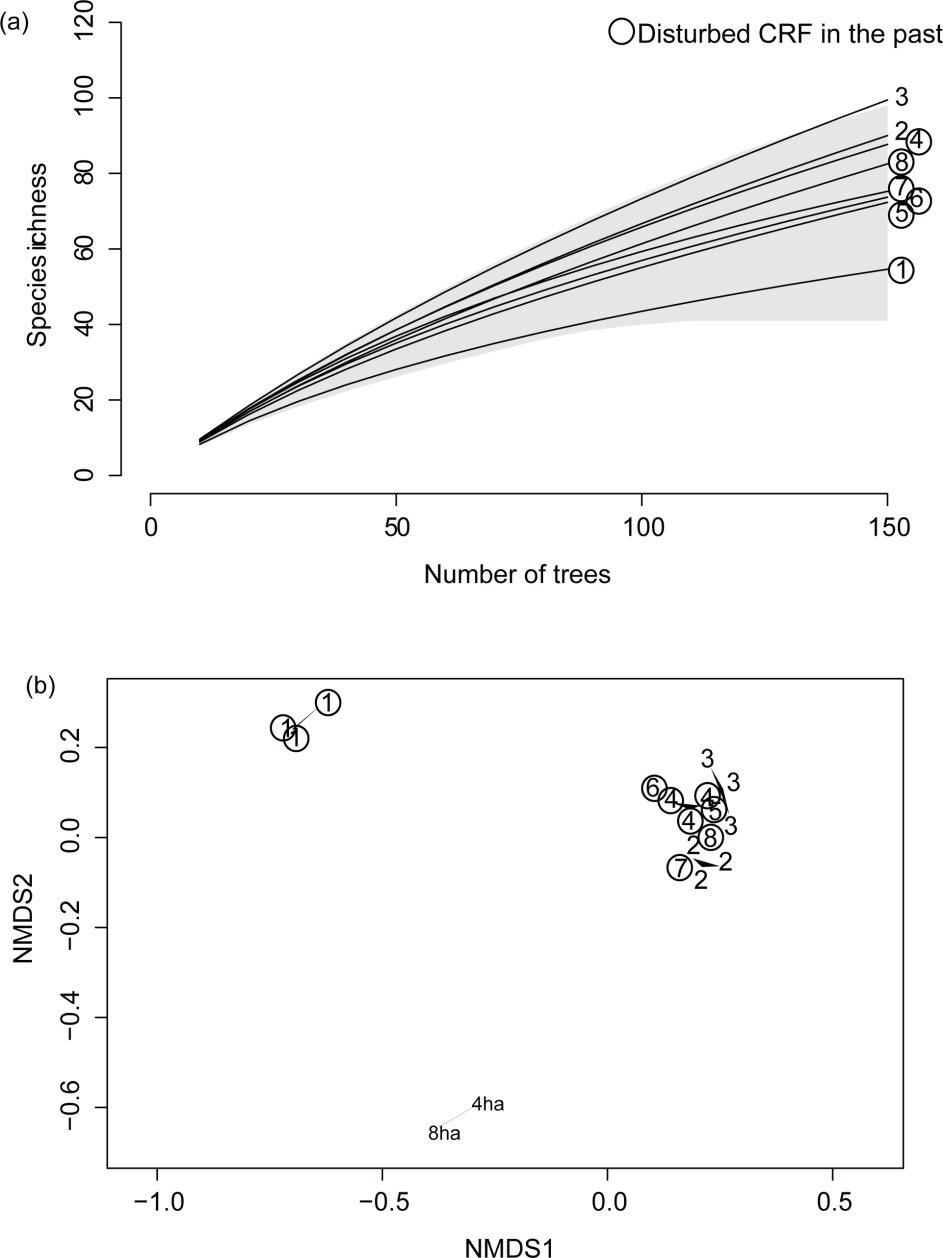
Alpha-diversity and composition of tree species communities in CRFs. (a) Sampling-standardized species richness within a 0.25-ha plot. Averaged rarefaction curves of eight CRFs are shown. The colored area indicates the 95% CI of LHNP. (b) NMDS ordination of tree species composition among 16 plots of eight CRFs and 8- and 4-ha plots in LHNP. Circles indicate the forests disturbed in the past. Plot replicates are linked with the lines.

We identified 2548 trees, which comprised 67 families, 183 genera, and 543 species in total (S1 Table, S2 Table). There were 14 trees which could not identify species, and we excluded them from further analysis. Tree communities in the Jelalong CRFs were mainly dominated by Dipterocarpaceae and Euphorbiaceae families (S2 Table), similar to those in LHNP (S3 Table). The lowest *S* (43) was observed in the mixed *kerangas* and peat swamp forest (CRF 1). *S* ranged from 74 to 97 in lowland or hill MDFs, which was equivalent to that of LHNP (Table 1). *H* was also the lowest in CRF 1 (approximately 3), whereas in the other forests it ranged from 3.9 to 4.2, which was similar to values of LHNP (Table 1). The rarefaction curves comparing CRFs and LHNP indicate that all CRFs had species diversity levels similar to the primary forest (Fig 2A). NMDS showed three clear clusters according to the region or soil type: LHNP, *kerangas*/peat swamp forest, and other MDFs in Jelalong (Fig 2B). In fact, 79% of species in CRF 1 are specific for this forest because this is the only *kerangas*/peat swamp forest among the target CRFs (S1 Table). In addition, 44% of the species in CRF 2 are also site-specific (S1 Table), which was the highest uniqueness among the MDF forests. There was no significant relationship between the *S*, *H*, or Simpson’s diversity index and the area of CRFs (generalized linear models, *p* > 0.10).

**Fig 3:**
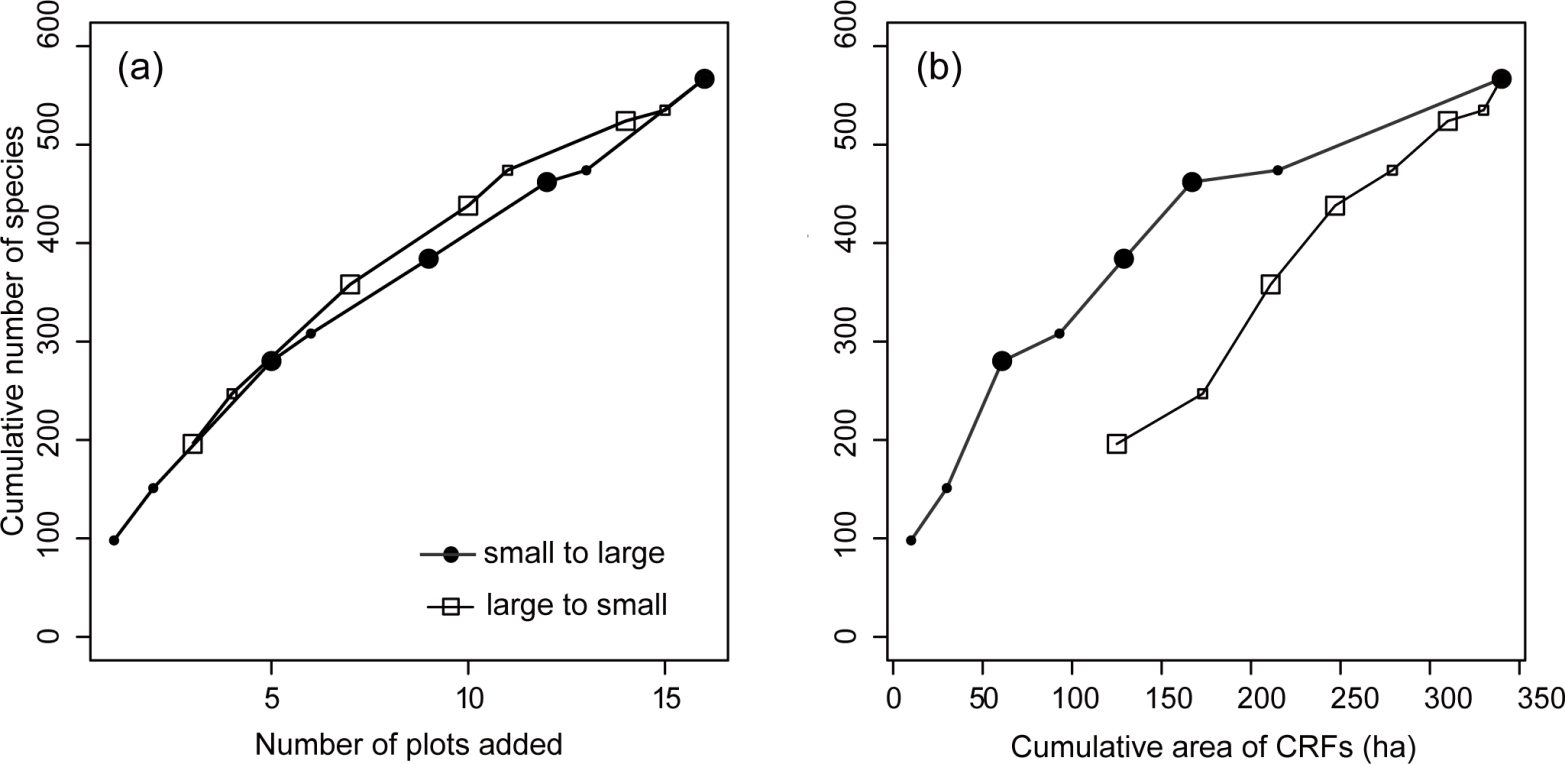
Species-area curve. Cumulative number of tree species sampled in 0.25-ha plots against (a) cumulative plot number and (b) CRF area added. The plots or CRFs were added from smallest to largest (black circle) or largest to smallest (blank square), respectively. The bigger symbols indicate that a CRF has three plots, i.e., three plots were added at once.

The cumulative *S* increased linearly as the plots were added, and the pattern was not very different whether the smallest or largest CRFs were added first (Fig 3A). The cumulative *S* against the cumulative area of CRFs increased largely when the smallest CRFs were added first than when the largest ones were added first (Fig 3B).

**Fig 4:**
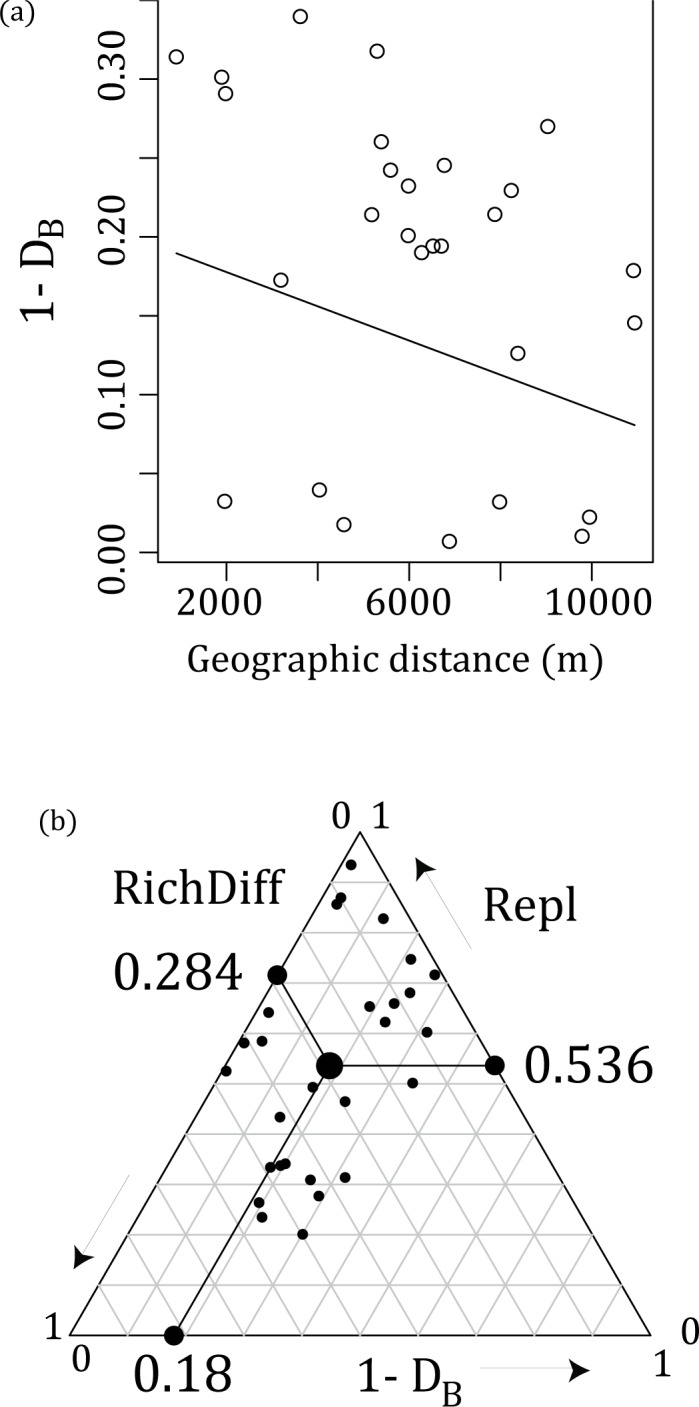
Beta-diversity of tree species communities in CRFs. (a) Distance-decay in the similarity (1-*D*_*B*_) of tree community composition among CRFs. The fitted model from a generalized linear model is shown in black. (b) Triangular graph of the relationship among 1-*D*_*B*_ (similarity), Repl (replacement), and RichDiff (richness difference). The large central dot is the centroid of the points, and the smaller dots represent the mean values of 1- *D*_*B*,_ *Repl*, and *RichDiff* components. The fractions of replacement and richness difference to the total beta diversity are *Repl*_*Prop*_ = 0.66 and *RichDiff*_*Prop*_ = 0.34, respectively.

We found a marginally significant negative geographic distance decay of similarity (1-*D*_*B*_; Mantel *r* = −0.29, *p* < 0.09) (Fig 4A). The triangle graph shows the similarity among CRFs and the components of replacement and richness difference (Fig 4B). The difference of tree species community was mostly because of replacement, which accounts for 65% of total beta diversity. Using *D*_*J*_, a similar tendency was observed (S1 Fig).

### Size structure

The size structure of the trees in Jelalong was different among CRFs (Table 1, S2 Fig). The percentage of trees with a DBH > 50 cm was > 6.5% in CRFs 1–3, which is almost equivalent to that in LHNP. Maximum values of DBH were also higher in those CRFs (> 90 cm). On the other hand, in the other CRFs, relatively small trees were predominant. In CRF 7, the mean DBH was significantly smaller than that in CRFs 1–3. Furthermore, CRF 7 contained smaller sized trees in a high density, which may be because this CRF experienced more intensive disturbance in the past compared with other disturbed CRFs.

### Species richness of endangered species

All CRFs contained endangered species according to the IUCN Red List [27] and Sarawak protected species [28] (Table 2). In total, 54 endangered or protected species were found in CRF plots. The number of endangered or protected species observed per CRF ranged from 5 to 24, and CRF 2–4 included > 17 species. We found no significant differences in the proportion of endangered species among CRFs (Fisher’s exact test, *p* > 0.05).

**Table 2:** Number of species in the IUCN Red List and Sarawak protected species.

CRF No.		1	2	3	4	5	6	7	8	Total
**IUCN redlist**^**1**^	**CR**	1	10	6	7	1	1	1	2	19
	**EN**	2	7	4	5	2	4	3	1	12
	**VU**	2	2	2	5	1	2	1	1	13
**Sarawak protected species**^**2**^		3	6	5	3	1	1	0	1	11
**CITES-Appendix II**		1	1	1	1	0	0	0	0	3
**Total**		8	24	17	20	5	8	5	5	54

1: IUCN Red List criteria: CR—Critically endangered, EN—Endangered, VU Vulnerable.

2: Sarawak protected species includes the species in the CITES list.

## Discussion

Our results showed that all CRFs harbor a high alpha and beta diversities, which suggests that all CRFs have a high conservation value. Although most CRFs experienced logging in the past, those CRFs had *S* and composition comparable to those of intact forests. This suggests that after disturbance, tree species diversity can recover because of seed dispersal from surrounding secondary forests. A previous study by Arroyo-Rodriguez, Pineda [26] showed that fragmented forests tend to have a higher species diversity if they have a high proportion of forest cover than those with a lower forest cover. Even if species diversity was decreased by human disturbance, immigration could compensate for the species loss. This could occur most effectively in landscapes with a higher forest cover. However, the size structure was different among disturbed and intact CRFs; disturbed CRFs still consist of relatively smaller trees. This suggests that biomass recovers more slowly than species diversity. These results are consistent with a previous study in Indonesian Borneo, which reported that conservative logging reduced the stem density, whereas *S* was equivalent with that of unlogged forest [29].

As for beta diversity, we found a marginally significant similarity in distance-decay, which could be caused by dispersal limitation or environmental heterogeneity, or both [30]. We also found a high beta diversity derived from a high turnover rate of species composition, not richness differences, among the sites. Thus, nestedness among CRFs is small, and it implies that all CRFs can be conservation targets. A high beta diversity has been reported in the region, which increases the regional diversity.

We also found that CRFs included a total of 54 species from the IUCN Red List species, Sarawak protected plant, or both. This indicates that CRFs in this small area (4 ha in total) could cover 17% of the IUCN Red List of Threatened species [categories of critically endangered (CR), endangered (EN), and vulnerable (VU)] among dicots (Magnoliopsida) occurring in Sarawak. Therefore, CRFs contribute to the conservation of endangered species. For example, endangered species, such as *ramin* (*Gonystylus* spp. Thymelaeaceae) and *gaharu* (*Aquilaria* spp., Thymelaeaceae), were found in CRFs. *Ramin* is a light hardwood, and it is a very valuable timber in the global market, whereas *gaharu* is used as an incense and in traditional medicines. As a high demand for both species in the global market has caused a reduction in their populations, they are now classified as VU in the IUCN Red List. Furthermore, timber export is restricted by CITES (listed in Appendix II) [31]. Even though *ramin* is valuable in the global market, local people seldom use these species by themselves. Because *ramin* contains an itchy resin, it is not a good timber for local use. Furthermore, local people used to use *gaharu* as medicines, but recently they prefer buying medicines from a drug store. Thus, villagers consume few *ramin* and *gaharu* specimens from CRFs, and therefore, CRFs could function as *in-situ* effective conservation spots for these species.

The high species diversity in CRFs maintained in Jelalong is probably owing to several specific reasons as big trees have rarely been cut down here in a long period at least 70 years after settlement of the longhouses. First, CRFs were traditionally considered as the timber stock for longhouse construction [16]. Second, accessing some CRFs was considered as a “taboo” because of spiritual beliefs [15]. Third, to maintain a constant supply and good quality of drinking water, CRFs that have a water catchment function are not supposed to be disturbed; however, this is a more recent trend. These customs may limit the disturbance of CRFs, resulting in a high biodiversity within the forests. A fourth reason could be that logging was not that intensive in the past, particularly in the steep terrain area. Furthermore, an ecological reason could be that CRFs are surrounded by old secondary forest (usually at least 25–40 years old after disturbance), which also functions as a species source. It has been previously argued that one of the important roles of small but intact forests in a fragmented landscape is to provide a seed source for surrounding degraded forests [16, 32]. The existence of these highly diverse forests can compensate for the species loss in degraded forests through seed dispersal [19]. The surrounding degraded forests can rehabilitate faster because of a constant seed input from small but intact forests, such as CRFs. Eventually, these forests will become a source of species for CRFs as well. Thus, if we can conserve the traditional mosaic landscape structure in the rural areas of Borneo, they might contribute to maintaining the species diversity at a regional level [33]. Future work might consider a comprehensive conservation of biodiversity, i.e., corridors between CRFs, particularly for vertebrate movement, which was also recommended by Ashton [13].

## Conclusions

CRFs can be a plausible biodiversity conservation target for the local government in the fragmented landscape of the Sarawak lowland area with respect to both alpha and beta diversity. Our results indicate that the traditional land use practice in indigenous communities, (i.e., CRFs) can be useful for biodiversity conservation, at least in the current situation. Furthermore, designing CRFs as a conservation target can be suitable for the maintenance of regional species diversity, including endangered species. However, long-term effective conservation might not be achieved without considering three points. First, the ecological and environmental factors that affect biodiversity in the long term are still unknown. Biodiversity dynamically changes in space and time in response to the physical environment, migration, and deterministic processes in plant demography [32, 34] as well as the surrounding land-use patterns [35, 36]. Second, local demand for CRFs, e.g., how much local people depend on products in CRFs and how people regard CRF functions, should be quantitatively evaluated in multiple aspects (e.g., [37]). Third, CRFs are prone to development under the pressure of economic incentives provided by the state and local governments and current trends in the global market. For example, one CRF in a village was partly opened by the villagers to plant oil palms because they believed that if they left their forest “unused,” the government or plantation companies may take the land. Consequently, CRFs have been decreasing in number and area because of socio-economic pressures, even though people have recently recognized their value. Biodiversity conservation using CRFs cannot be achieved without reducing the pressure of land development. Thus, it is essential that local communities, companies, and policy makers are together engaged in implementing a conservation strategy that is integrated with their land-use practice.

## Supporting information

S1 Table Number of taxonomic classification of tree species community in each CRF. (PDF)

S2 Table Taxonomic classification, abundance and conservation status of tree species community in each CRF. (XLSX)

S3 Table Taxonomic classification, abundance of tree species community in 4ha (2000) and 8ha (1997) plots in Lambir Hills National Park. (XLSX)

S1 Fig Beta-diversity of tree species communities in CRFs. (a) Distance-decay in the similarity (1-*D*_*J*_) of tree community composition among CRFs. The fitted model from a generalized linear model is shown in black. (b) Triangular graph of the relationship among 1-*D*_*J*_ (similarity), Repl (replacement), and RichDiff (richness difference). The large central dot is the centroid of the points, and the smaller dots represent the mean values of 1- *D*_*J*,_ *Repl*, and *RichDiff* components. The fractions of replacement and richness difference to the total beta diversity are *Repl*_*Prop*_ = 0.62 and *RichDiff*_*Prop*_ = 0.26, respectively.
(EPS)

S2 Fig DBH size frequency of each CRF in jalalong. Means followed by the same letter are not significantly different at the 0.05 level by pairwise *t* test adjusted using false discovery rate.
(EPS)
