# Supplementary material for: Biodiversity conservation values of fragmented communally reserved forests, managed by indigenous people, in a human-modified landscape in Borneo
Source: PLoS One. 2017 Nov 29;12(11):e0187273. doi: 10.1371/journal.pone.0187273 (PMC5706675; doi:10.1371/journal.pone.0187273)
Supplement: S1 Table — (PDF) [file pone.0187273.s001.pdf]

S1 Table. Number of taxonomic classification of tree species community in each CRF

**CRF No.**	1	2	3	4	5	6	7	8
Family	35	49	39	46	31	27	33	29
Genus	60	105	100	95	54	52	70	54
Species	91	189	180	184	79	88	97	74
Unique species	72	83	58	39	8	17	30	9
Proportion of unique species	0.79	0.44	0.32	0.21	0.10	0.19	0.31	0.12
